# Olfactory Dysfunction and Its Association With Neuropathologic Proteins in Cerebrospinal Fluid From Patients With Parkinson Disease

**DOI:** 10.3389/fnagi.2020.594324

**Published:** 2020-12-11

**Authors:** Peng Guo, Rui-dan Wang, Teng-hong Lian, Du-yu Ding, Ya-nan Zhang, Wei-Jiao Zhang, Dan-ning Li, Li-xia Li, Jing-hui Li, Hui-ying Guan, Shu-yang Yu, Li Liu, Yang Hu, Li-jun Zuo, Qiu-jin Yu, Xiao-min Wang, Wei Zhang

**Affiliations:** ^1^Department of Neurology, Beijing Tiantan Hospital, Capital Medical University, Beijing, China; ^2^Department of Blood Transfusion, Beijing Tiantan Hospital, Capital Medical University, Beijing, China; ^3^Department of General Internal Medicine, Beijing Tiantan Hospital, Capital Medical University, Beijing, China; ^4^Department of Physiology, Capital Medical University, Beijing, China; ^5^Department of Neurology, Center for Cognitive Neurology, Beijing Tiantan Hospital, Capital Medical University, Beijing, China; ^6^China National Clinical Research Center for Neurological Disease, Beijing Tiantan Hospital, Capital Medical University, Beijing, China; ^7^Center of Parkinson's Disease, Beijing Institute for Brain Disorders, Beijing, China; ^8^Beijing Key Laboratory on Parkinson Disease, Beijing, China

**Keywords:** Parkinson disease, olfactory dysfunction, sniffin' sticks test, cerebrospinal fluid, neuropathologic proteins

## Abstract

**Background and Purpose:** Olfactory dysfunction (OD) is a common non-motor symptom of Parkinson disease (PD). However, the relationship between OD and neuropathologic proteins in cerebrospinal fluid (CSF) from PD patients remains unclear.

**Methods:** 166 PD patients were included in the study. Overall olfactory function was assessed by summing up the scores of olfactory threshold, discrimination, and identification by a Sniffin' Sticks test, based on which, patients were divided into PD with OD (PD-OD) and PD with no OD (PD-NOD) groups. CSF samples were obtained from 76 PD patients. The levels of neuropathologic proteins, including α-Synuclein, Aβ1-42, total tau (T-tau), and multiple forms of phosphorylated tau (P-tau) in CSF were measured by an enzyme-linked immunosorbent assay.

**Results:** out of the 166 PD patients, 103 cases (62.0%) had OD. The scores of overall olfactory functions, and olfactory threshold, discrimination, and identification in the PD-OD group were all significantly lower than that in the PD-NOD group (*P* < 0.001). α-Synuclein level in CSF was significantly higher in the PD-OD group than the PD-NOD group (*P* < 0.05), and was significantly and negatively correlated with the scores of overall olfactory function, and olfactory discrimination and identification (*P* < 0.05). Aβ1-42 level in CSF was higher in the PD-OD group than the PD-NOD group, and was significantly and negatively correlated with the olfactory identification score (*P* < 0.05). T-tau level in CSF was significantly lower in the PD-OD group than the PD-NOD group (*P* < 0.05), and was significantly and positively correlated with the olfactory discrimination score (*P* < 0.05). There was no significant difference in P-tau level in CSF between the PD-OD and PD-NOD groups and no correlation between OD score and P-tau level in CSF.

**Conclusions:** PD-OD includes the impairments of olfactory threshold, discrimination, and identification, and is associated with the significant elevation of α-Synuclein and the decrease of the T-tau level in CSF.

## Background

A pathological hallmark of Parkinson disease (PD) is Lewy bodies (LBs), which deposit in the substantia nigra (SN) and cause the progressive degeneration and death of dopaminergic neurons, leading to dopamine depletion in striatum and subsequent motor symptoms. Recent pathological investigations demonstrated that LBs beyond SN were associated with numerous non-motor symptoms (Khoo et al., 2013), including neuropsychiatric symptoms, autonomic dysfunction, sleep disorders, and abnormal sensation, etc., among which, olfactory dysfunction (OD) was a common non-motor symptom of abnormal sensation. The high prevalence of OD, along with the low cost of olfactory tests, has fostered great interest in olfaction as a potential biomarker for PD (Fullard et al., 2017).

According to the Braak stage of PD pathology (Braak et al., 2003), LBs in the brain start firstly in the olfactory bulb and anterior olfactory nucleus, and subsequently in nuclei in the lower brainstem, advancing in a topographically predictable sequence, to the upper brainstem and finally to the cerebral cortex. Thus, theoretically, OD is supposed to be the first symptom of PD (Haehner et al., 2007), indicating that it appears prior to motor symptoms (Ponsen et al., 2010). Currently, OD is considered as an indicator of the preclinical or early stage of PD (Doty, 2012).

α-Synuclein is the major component of LBs when it is excessively aggregated, genetically mutated, or abnormally modified. Compared with patients with OD caused by other diseases, no specific changes were found in the olfactory epithelium of the nasal cavity in PD patients, indicating that pathological changes in the brain instead of a peripheral organ were associated with PD with OD (PD-OD) (Witt et al., 2009). A body of evidence revealed the relevance between OD and α-Synuclein in PD brains. For example, the first deposit of LBs in the olfactory bulb (Ferrer et al., 2012) based on the Braak stage of PD pathology indicated an association between PD-OD and α-Synuclein. Furthermore, LBs were observed in the piriform cortex of postmortem PD brains (Silveira-Moriyama et al., 2009). Additionally, it was found that LBs in SN and the limbic system were also related to PD-OD (Wilson et al., 2011). However, the α-Synuclein level in cerebral spinal fluid (CSF) from PD-OD patients and its correlation with PD-OD have not been reported.

β amyloid (Aβ) is the major component of neuritic plaque, a pathological biomarker of Alzheimer's disease (AD). In histologically diagnosed PD patients, Aβ was frequently observed in the brain, suggesting its relationship with PD11. Another investigation showed that Aβ1-42 level in CSF was significantly lowered in those with worse OD identified by the University of Pennsylvania Smell Identification Test (UPSIT) (Fullard et al., 2016). However, it failed to explore the correlation between Aβ1-42 level in CSF and each type of OD, including olfactory threshold (THR), discrimination (DIS), and identification (ID) in PD patients (Fullard et al., 2016).

Tau is a microtubule-associated protein. Over-phosphorylated tau (P-tau) is the major component of neurofibrillary tangle, another pathological hallmark of AD. When tau is over-phosphorylated, it leads to the degeneration and death of neurons (Goris et al., 2007). Tau pathology is also present in the olfactory bulb of PD patients, demonstrating a correlation between tau and PD-OD (Mundinano et al., 2011). It was considered that gene-related influences on PD-OD, when present, needed time to develop and depended upon additional factors, such as tau-related pathology (Doty, 2012). However, the correlation between the levels of tau pathology (T-tau and multiple forms of phosphorylated tau (P-tau) in CSF) and PD-OD are poorly understood.

Based on above status, this study aimed to investigate the correlation between the levels of neuropathologic proteins, including α-Synuclein, Aβ1-42, T-tau, and multiple forms of P-tau in CSF and olfactory function, including olfactory THR, DIS, and ID in PD patients.

## Methods

We conducted a cross-sectional study. The study was approved by the ethics review board of Beijing Tiantan Hospital, Capital Medical University, and written informed consent was obtained from all 166 PD patients consecutively recruited from this hospital.

### Subjects

#### Inclusion Criteria for PD

Clinically established and probable PD patients were diagnosed according to the diagnostic criteria of PD published by the Movement Disorder Society (MDS) in 2015 (Postuma et al., 2015).

#### Exclusion Criteria for Participants

The exclusion criteria for participants included: (1) family history of OD. Olfactory disorders can be inherited. For example, ciliary disease is a type of inherited pleiotropic genetic disease. The changes in the assembly, maintenance, and/or function of cilia are manifested in multiple organ systems. Anosmia is a clinical manifestation that has been confirmed in patients (Uytingco et al., 2019). Therefore, hereditary olfactory disorders caused by non-PD diseases may aggravate the degree of olfactory dysfunction in individuals with PD, so that it cannot truly reflect the real cause of a patient's olfactory disorder, which may be caused by PD pathology and genetic factors. This may exaggerate the smell of PD patients. (2) History of drug abuse; long-time exposure to volatile substances or special environments, including herbicides, pesticides, metal dust, acidic gases, industrial thinners, detergents, sawdust, and mining areas; (3) illness or surgical history of the nasal cavity or sinus; (4) respiratory infections, such as rhinitis, bronchitis, and pneumonia, etc., within one month; (5) chronic obstructive pulmonary diseases; (6) other neuropsychiatric disorders, such as AD, schizophrenia, epilepsy, and multiple sclerosis, etc., that affect olfactory function; (7) history of head trauma; (8) infections, allergies, endocrine disorders, and autoimmune diseases; and (9) in menstruation or pregnancy.

Demographic variables, including gender, age, age of onset, disease duration, educational level, smoking, and medications used, etc., were recorded. Particularly, the daily dose of anti-PD drugs, including levodopa, dopamine receptor agonist, monoamine oxidase-B inhibitor, catechol-oxygen-methyltransferase inhibitor, and amantadine, etc., were collected. Levodopa equivalent daily doses (LEDD) were calculated as follows: LEDD = levodopa standard tablets × 1 + levodopa controlled-release tablet × 0.75 + (levodopa standard tablets × 1 + levodopa controlled-release tablet × 0.75) × 0.25 (if taking entacapone simultaneously) + piribedil sustained-release tablets × 1 + pramipexole hydrochloride tablet × 100 + selegiline hydrochloride tablets × 10.

### Evaluation of Olfactory Function

The olfactory function of PD patients was tested by Sniffin' Sticks from the Burghart Messtenik Company, German (product number: LA-13-00005). A total of 112 sticks were used for the test, in which 48, 48, and 16 were used for olfactory THR, DIS, and ID, respectively.

Olfactory THR: participants were exposed to n-butyl alcohol from the lowest concentration to the highest one. The THR score was the number of Sniffin' Sticks that participants clearly identified with the minimal concentration of n-butyl alcohol. The lower the THR score, the worse the olfactory recognition.

Olfactory DIS: participants were instructed to figure out the target odor that was different from the other two. The DIS score was the number of Sniffin' Sticks that participants correctly identified. The lower the DIS score, the worse the olfactory discrimination.

Olfactory ID: participants were required to smell odor and choose the right answer from the given four. The ID score was the number of Sniffin' Sticks that participants correctly identified with the target odor. The lower the ID score, the worse the olfactory identification.

Overall olfactory function was obtained by summing up the scores of THR, DIS, and ID (TDI).

OD was identified by the following criteria from a cross-sectional study of 3,282 people by a Sniffin' Sticks test adjusted for sex and gender (Hummel et al., 2007). OD was established if an individual was between 36 and 55 years old, male with a TDI score <24.95, or female with a TDI score <28.75; if an individual was >55 years old, male with a TDI score <19.75, or female with a TDI score <19.05.

A total of 166 patients completed the Sniffin' Stick test, among which, 103 cases were divided into the PD-OD group, and 63 cases were put into the PD with no OD (PD-NOD) group.

### Collection of CSF Samples

CSF samples were obtained from 76 PD patients, including 35 males and 41 females, of which demographic variables were also collected.

Anti-PD drugs were withheld for 12–14 h if the patient's condition allowed. In order to prevent the blood contamination of CSF, the lumber puncture for each patient was performed by a professionally trained neurologist, strictly following the standardized protocol. The first and second tubes may contain blood, as well as tissue fragments and contaminated skin microorganisms, thus, routinely, the third tube of CSF was retained to ensure that the CSF was not contaminated by blood to an acceptable degree. CSF samples from both the PD-OD and PD-NOD groups in this study all followed this standardized protocol, trying to avoid this potential bias. A total of 5 ml CSF was obtained through lumbar puncture in polypropylene tubes between 7-10 a.m. under fasting conditions. CSF samples were centrifuged immediately at 3,000 rpm at 4°C. Approximately 0.5 ml supernatant of CSF was aliquoted into separate Nunc cryotubes and kept frozen at −80°C until ready for assay. Each aliquot dedicated for each measure to avoid freeze-thawing and protein degradation.

### Measurements of Neuropathologic Proteins in CSF

The levels of neuropathologic proteins in CSF from PD patients were measured using an enzyme-linked immunosorbent assay. We used the Human α-Synuclein (SNCA) ELISA Kit (Catalog Number: CSB-E18033h) produced by CUSABIO. According to the contents of the instructions and the company's response, this kit is designed for the full-length sequence of α-Synuclein. Therefore, it can both measure monomers and complexes, that is, total α-Synuclein was measured in this study. The CSB-E12011h Kit for T-tau was obtained from the Huamei Bioengineer Company (Wuhan, China). The Invitrogen-KHB8051 Kit for P-tau (T231), Invitrogen-KHB7041 Kit for P-tau (S199), Invitrogen-KHB7031 Kit for P-tau (S396), and Invitogen-KHO0631 Kit for P-tau (T181) were all obtained from the YuBo Bioscience Co. Ltd (Shanghai, China).

### Data Analyses

Statistical analyses were performed with SPSS Statistics 22.0 (IBM Corporation, New York, USA). *P* < 0.05 was statistically significant.

Demographic variables, the scores of olfactory THR, DIS, and ID, and the levels of neuropathologic proteins, including α-Synuclein, Aβ1-42, T-tau, and multiple forms of P-tau in CSF were compared between the PD-OD and PD-NOD groups.

Continuous variables, if they were normally distributed, were presented as means ± standard deviations and compared by a two-tailed *t*-test. Bonferroni correction was performed in further comparisons between the PD-OD and PD-NOD groups. Continuous variables, if they were not normally distributed, were presented as median (quartile) and compared by a nonparametric test.

Discrete variables were presented as percentages, and a Chi square test was used to make comparisons.

Spearman correlation analyses were made between the scores of TDI, THR, DIS, and ID and the levels of neuropathologic proteins in CSF.

## Results

### Frequency of PD-OD

Firstly, 166 PD patients were required to describe the status of their olfactory function, among which, 79 cases (47.6%) reported that they had OD.

Secondly, the olfactory function of each PD patient was tested by a Sniffin' Sticks test. It was found that 103 out of the 166 cases (62.0%) actually had OD.

Finally, in 103 PD patients with OD demonstrated by the Sniffin' Sticks test, 63 cases (61.2%) had OD before the occurrence of motor symptoms.

### Demographic Variables of the PD-OD and PD-NOD Groups

Demographic variables of the PD-OD and PD-NOD groups were compared (Table 1) and no significant differences in gender, age, age of onset, educational level, smoking rate, and LEDD were found.

**Table 1:** Demographic variables of the PD-OD and PD-NOD groups.

		**PD-NOD group**	**PD-OD group**	***p***
		**(*n* = 63)**	**(*n* = 103)**	
Gender				0.14
	Male [case (%)]	25 (39.7%)	53 (51.5%)	
	Female [case (%)]	38 (60.3%)	50 (48.5%)	
Age [years, (mean ± SD)]		63.00 ± 12.46	62.75 ± 9.98	0.891
Age of onset [(mean ± SD)]		59.07 ± 12.96	58.25 ± 10.44	0.664
Disease duration [years, median (Q1, Q3)]		3.0 (1.0, 5.0)	3.0 (2.0, 5.0)	0.281
Educational level [case (%)]				0.415
	Primary school and below	25 (39.7%)	50 (48.5%)	
	Middle school	26 (41.3%)	41 (39.8%)	
	College and above	12 (19.0%)	12 (11.7%)	
Smoking rate [case (%)]		22 (34.9%)	45 (43.7%)	0.264
LEDD [mg, median (Q1, Q3)]		250 (0.0, 475.0)	300 (0.0, 568.8)	0.330

*SD, standard deviation; Q1, first quartile; Q3, third quartile; PD-OD, Parkinson disease with olfactory dysfunction; PD-NOD, Parkinson disease with no olfactory dysfunction; LEDD, levodopa equivalent daily dose*.

### Clinical Features of OD in PD Patients

In PD patients, the TDI score was 18.99 ± 5.87 points, in which the THR score was 3.50 (2.00, 5.00) points, the DIS score was 8.00 (6.00, 10.00) points, and the ID score was 7.00 (5.00, 9.00) points.

Further comparison analyses revealed that the TDI score as well as the scores of THR, DIS, and ID were all significantly lower than that in the PD-NOD group (**Table 3**).

### Relationship Between Neuropathologic Proteins in CSF and Olfactory Function in PD Patients

The levels of neuropathologic proteins, including α-Synuclein, Aβ1-42, T-tau, and multiple forms of P-tau in CSF from 76 patients were measured, among which, 52 and 24 cases were from the PD-OD and PD-NOD group, respectively.

Firstly, α-Synuclein levels of the PD-OD and PD-NOD groups were compared (**Table 3**). It was found that the α-Synuclein level in CSF from the PD-OD group was significantly higher than that from the PD-NOD group, indicating that the elevation of α-Synuclein in CSF was related to PD-OD. Further analysis indicated that α-Synuclein level in CSF from PD patients was significantly and negatively correlated with TDI, DIS, and ID scores, but not with THR score (**Table 4**), indicating that OD, especially DIS and ID, deteriorated as the α-Synuclein level in CSF rose.

Secondly, the levels of Aβ1-42 in CSF in the PD-OD and PD-NOD groups were compared (**Table 3**). It was observed that the Aβ1-42 level in CSF from the two groups were not significantly different. Further analysis implied that the Aβ1-42 level in CSF had a significantly negative correlation with the ID score (**Table 4**), suggesting that ID worsened as Aβ1-42 level in CSF increased in PD patients.

Thirdly, the levels of T-tau and P-tau in CSF from the PD-OD and PD-NOD groups were compared (**Table 3**). It was revealed that the T-tau level in CSF from the PD-OD group was significantly decreased when compared with that from the PD-NOD group, implying that the decrease of T-tau in CSF was associated with PD-OD. The levels of P-tau (S199), P-tau (S396), P-tau (T181), and P-tau (T231) between the two groups exhibited no significant differences. Thus, T-tau was associated with PD-OD. Further analysis revealed that the T-tau level in the CSF of PD patients had a significantly positive correlation with DIS score (**Table 4**), indicating that DIS progressed as T-tau level in CSF declined.

**Figure 1:**
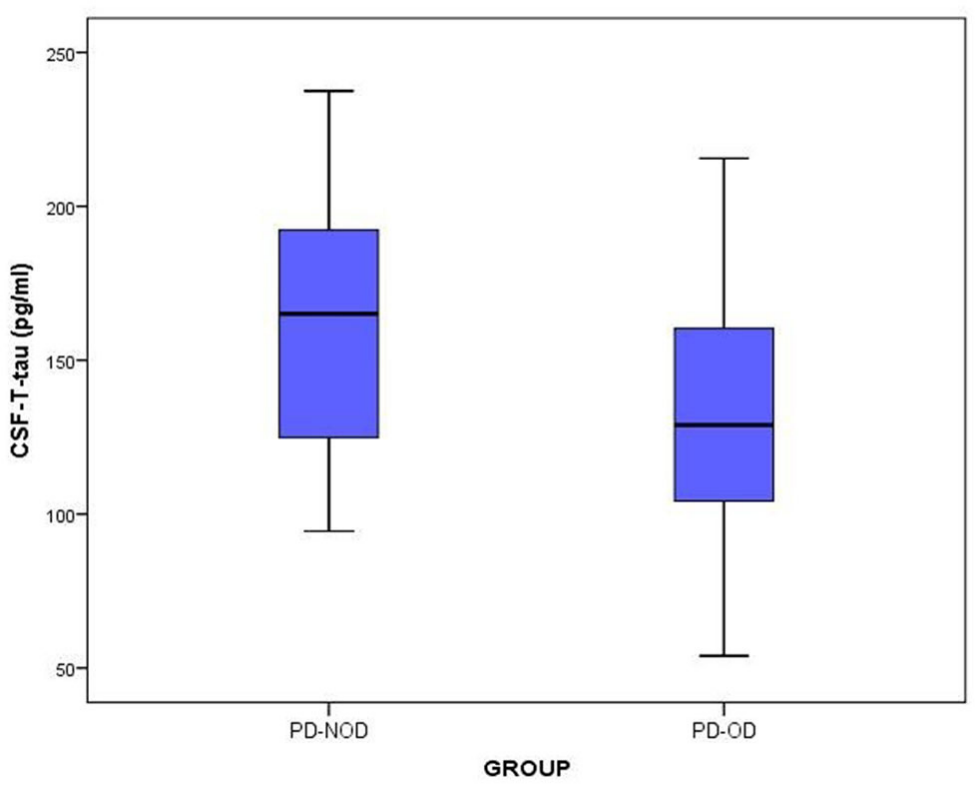
The levels of T-tau in CSF from PD-OD and PD-NOD groups. T-tau, total tau; PD-OD, Parkinson disease with olfactory dysfunction; PD-NOD, Parkinson disease with no olfactory dysfunction.

**Figure 2:**
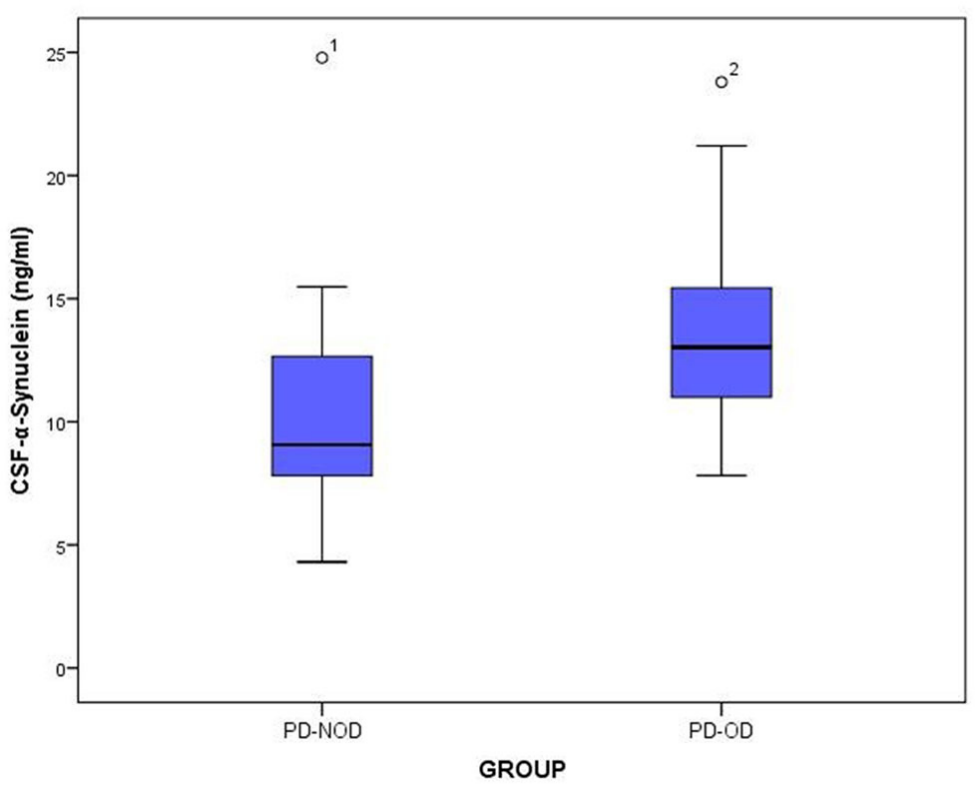
The levels of a-Synuclein in CSF from PD-OD and PD-NOD groups. PD-OD, Parkinson disease with olfactory dysfunction; PD-NOD, Parkinson disease with no olfactory dysfunction.

**Figure 3:**
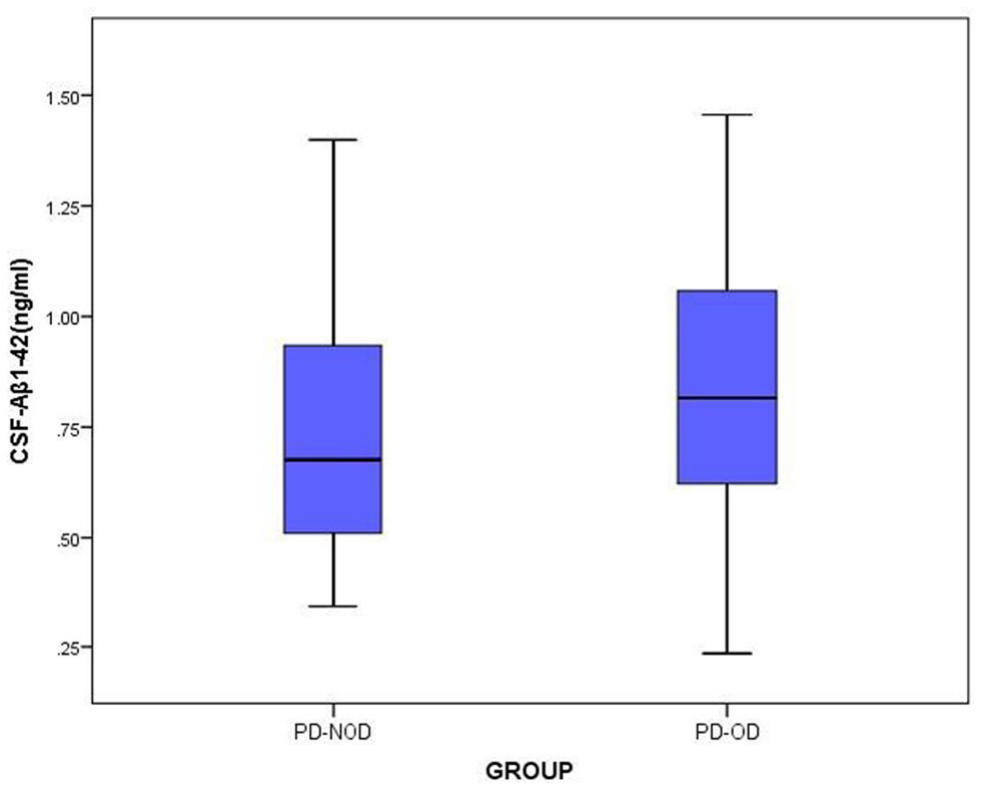
The levels of Aβ1-42 in CSF from PD-OD and PD-NOD groups. PD-OD, Parkinson disease with olfactory dysfunction; PD-NOD, Parkinson disease with no olfactory dysfunction.

The total tau and α-Synuclein in CSF showed a significant difference between the PD-OD and PD-NOD groups, but there appeared to be significant overlap. We can see this overlap using a box and whiskers plot (Figures 1–3).

## Discussion

In recent years, OD, a common non-motor symptom of PD, has become an important research topic. Currently, it is widely accepted that OD may play a pivotal role in predicting and indicating the early development of PD.

In this investigation, the percentage of OD reported by the PD patients was 47.6% (79/166 cases). However, this percentage went up to 62.0% (103/166 cases) when olfactory function was objectively tested by Sniffin' Sticks and determined by the criteria adjusted for gender and sex (Haehner et al., 2009). Although people with PD are aware of hyposmia to some extent, individuals often overestimate their ability to smell, making self-report an unreliable source of information (Leonhardt et al., 2019). Olfactory function is vulnerable to multiple factors. For example, most PD patients are elderly, so they are not sensitive to olfactory alterations. Additionally, the decline of olfactory function may not be evident at an early stage of PD and thus is easily ignored by patients. Accordingly, a sensitive and objective approach should be used for evaluating olfactory function and diagnosing PD patients with OD. In a previous study by Haehner, the mean disease duration was found to be 6.6 years in 400 recruited PD patients (Haehner et al., 2009), whereas, it was 3.0 years in the collected 166 PD patients in this study. The significantly shortened disease duration may explain the lower ratio of PD-OD in our study. In the study of Mahlknecht (Mahlknecht et al., 2016), 134 patients with PD and 46 patients with atypical Parkinsonism (23 cases of multiple system pathology, 23 cases of progressive supramolecular palsy) were recruited, which is different from the pure PD patients recruited in our study. Therefore, this may account for the different ratio of PD-OD between the two studies.

It is important to note that, in 103 PD patients with OD identified by Sniffin' Sticks, 63 cases (61.2%) had significant olfactory function impairment before the occurrence of motor symptoms, illustrating that OD is a common non-motor symptom of PD prior to movement dysfunction (Pont-Sunyer et al., 2015). LBs are firstly formed in the olfactory bulb and anterior olfactory nucleus, hence, theoretically, OD may be the first symptom of PD (Haehner et al., 2007). Clinically, doctors should evaluate olfactory function with an objective approach for the population with a high risk of PD, and provide valuable cues for the early diagnosis of PD.

By comparing demographic variables, no significant difference was found between the PD-OD and PD-NOD groups (Table 1), indicating that gender, age, age of onset, educational level, smoking rate, and dopaminergic drugs were not involved in PD-OD.

The above demographic variables were also compared between the PD-OD and PD-NOD groups for the subjects with CSF samples collected (Table 2). Although the results also indicated no significant differences, patients who had CSF samples collected seemed to be slightly younger, had a marginally earlier onset of age, a shorter disease duration, and lower LEDD, implying that younger patients and those in the early stage of PD preferred to accept an invasive lumber puncture, while the older and severer PD patients had more difficulties in completing it.

**Table 2:** Demographic variables of patients with CSF in the PD-OD and PD-NOD groups.

		**PD-NOD group**	**PD-OD group**	***p***
		**(*n* = 24)**	**(*n* = 52)**	
Gender				0.131
	Male [case (%)]	8 (33.3%)	27 (51.9%)	
	Female [case (%)]	16 (66.7%)	25 (48.1%)	
Age [years (mean ± SD)]		60.00 ± 12.27	60.69 ± 11.65	0.743
Age of onset [(mean ± SD)]		56.70 ± 12.33	57.40 ± 11.74	0.814
Disease duration [years, median (Q1, Q3)]		2.75 (1.0, 4.0)	3.0 (1.32, 4.75)	0.577
Educational level [case (%)]				0.821
	Primary school and below	11 (45.8%)	27 (52.0%)	
	Middle school	9 (37.5%)	19 (36.5%)	
	College and above	4 (16.7%)	6 (11.5%)	
Smoking rate [case (%)]		7 (29.2%)	23 (44.2%)	0.212
LEDD [mg, median (Q1, Q3)]		100 (0.0, 393.75)	287.5 (0.0, 487.5)	0.652

*SD, standard deviation; Q1, first quartile; Q3, third quartile; PD-OD, Parkinson disease with olfactory dysfunction; PD-NOD, Parkinson disease with no olfactory dysfunction; CSF, cerebrospinal fluid; LEDD, levodopa equivalent daily dose*.

In this investigation, olfactory TDI score and the scores of olfactory THR, DIS, and ID in the PD-OD group were all significantly lower than that in the PD-NOD group (Table 3). A previous study reported that OD manifested as comprehensive impairments of olfactory THR, DIS, and ID, in which, olfactory DIS and ID impairments occurred earlier and were more severe (Doty, 2012). However, another investigation showed that olfactory DIS and ID were evidently compromised while olfactory THR was unchanged in some PD patients (Whitcroft et al., 2017). The results from the investigations above varied, which might be accounted for the differences in the stage and duration of PD patients and the methods of evaluating olfactory function.

**Table 3:** Olfactory function between the PD-OD and PD-NOD groups.

	**PD-NOD group**	**PD-OD group**	***p***
	**(*n* = 63)**	**(*n* = 103)**	
Olfactory TDI [point, (mean ± SD)]	24.11 ± 3.92	16.10 ± 4.71	<0.001^**^
THR [point, median (Q1, Q3)]	5.0 (3.5, 6.0)	3.0 (2.0, 4.0)	<0.001^**^
DIS [point, median (Q1, Q3)]	10.0 (9.0, 11.0)	7.0 (5.0, 9.0)	<0.001^**^
ID [point, median (Q1, Q3)]	9.0 (7.0, 10.5)	6.0 (4.5, 7.0)	<0.001^**^

** *P < 0.01; TDI, threshold, discrimination and identification; THR, threshold; DIS, discrimination; ID, identification; SD, standard deviation; Q1, first quartile; Q3, third quartile; SD, standard deviation; PD-OD, Parkinson disease with olfactory dysfunction; PD-NOD, Parkinson disease with no olfactory dysfunction*.

α-Synuclein consists of 140 amino acids and α-Synuclein-containing LBs are a pathological hallmark of PD. It was reported that the total α-Synuclein level declined, while the α-Synuclein oligomer level was elevated in CSF from PD patients (Parnetti et al., 2014). The Braak stage of PD pathology revealed a close correlation between OD and a deposit of α-Synuclein. However, no investigation focused on the change of α-Synuclein in CSF from the PD-OD population. In this study, the α-Synuclein level in CSF from the PD-OD group was significantly higher than that in the PD-NOD group (Table 4), indicating a massive deposition and release of α-Synuclein from olfaction-related brain regions to CSF. Under physiological conditions, α-Synuclein plays a role in regulating the release of neurotransmitters and maintaining synaptic plasticity without damaging neurons. However, when it is misfolded, the formed pathological α-Synuclein or Lewy bodies (LBs) exert a neurotoxic effect on neurons. The pathological aggregation of α-Synuclein and the inflammatory response interact with each other, that is, the abnormal aggregation of α-Synuclein can cause neuroinflammation, and the neuroinflammatory response can further accelerate the abnormal aggregation of α-Synuclein (Caggiu et al., 2019). This vicious cycle may result in a large amount of α-Synuclein accumulation in the olfactory bulb. A variety of nerve cells in the brain, including neurons, astrocytes, microglia, etc., can eliminate extracellular α-Synuclein through endocytosis, but this process has not been fully elucidated. Both *in vivo* and *in vitro* studies have shown that toll-like receptors (TLRs) have different phagocytosis effects on different forms of α-Synuclein. TLR2 in microglia can bind with α-Synuclein oligomers and phagocytize them (Kim et al., 2013), while TLR4 is necessary for the phagocytosis of fibrous α-Synuclein, and the lack of TLR4 can impair the endocytosis of microglia, decreasing the clearance of α-Synuclein and aggravation of neurodegeneration (Stefanova et al., 2011). Activated microglia significantly reduce the efficiency of α-Synuclein degradation (Lee et al., 2008; Stefanova et al., 2011). In addition, astrocytes can degrade α-Synuclein through the lysosomal pathway after taking up α-Synuclein, but when the uptake of α-Synuclein by astrocytes exceeds a certain amount, lysosomal metabolism is restricted (Lindstr m et al., 2017). Therefore, at the early stage of neuroinflammation, glial cells benefit from clearing α-Synuclein. As time goes by, the impaired physiological functions of glial cells reduce α-Synuclein clearance, and thus elevate the α-Synuclein level, causing the progression of PD. In this study, the PD-OD group had a longer course of disease compared with the PD-NOD group (averagely, 3 vs. 1 years), which may explain the significantly elevated α-Synuclein level in CSF due to the compromised physiological function of glial cells, and subsequently eliminated α-Synuclein clearance.

**Table 4:** The levels of neuropathologic proteins in CSF from the PD-OD and PD-NOD groups.

	**PD-NOD group**	**PD-OD group**	***p***
	**(*n* = 24)**	**(*n* = 52)**	
α-Synuclein (ng/ml, mean ± SD)	10.08 ± 3.22	13.55 ± 3.93	<0.001^*^
Aβ1-42 (ng/ml, mean ± SD)	0.74 ± 0.28	0.84 ± 0.34	0.23
T-tau (pg/ml, mean ± SD)	163.93 ± 42.99	138.33 ± 40.30	0.031
P-tau (S199) (pg/ml, mean ± SD)	10.65 ± 4.25	11.06 ± 7.03	0.802
P-tau (S396) (pg/ml, mean ± SD)	72.50 ± 29.81	71.02 ± 26.01	0.834
P-tau (T181) (pg/ml, mean ± SD)	67.33 ± 31.54	74.24 ± 30.13	0.384
P-tau (T231) (pg/ml, mean ± SD)	147.83 ± 68.22	174.97 ± 85.88	0.207

* *P was significant after Bonferroni correction. Aβ, β amyloid; T-tau, total tau; P-tau, phosphorylated tau; SD, standard deviation; Q1, first quartile; Q3, third quartile; PD-OD, Parkinson disease with olfactory dysfunction; PD-NOD, Parkinson disease with no olfactory dysfunction*.

Parnetti et al. (Parnetti et al., 2019) pointed out that the decreased total α-Synuclein level in CSF can be used as a marker of Synuclein-opathy, and the increased α-Synuclein level can be used as a non-specific marker of synaptic damage. In addition, PD patients with high levels of total α-Synuclein in CSF have a faster decline in motor function, implying that the increased total α-Synuclein level in CSF may also predict the adverse outcome of PD patients. Therefore, the dual changes of total α-Synuclein level in CSF increases the complexity of analyzing and interpreting its results. Hence, total α-Synuclein level in CSF does not seem to be a reliable diagnostic marker for PD, while a study focusing on the more pathophysiological-specific species of α-Synuclein, and the combination of total α-Synuclein with other biomarkers in CSF may provide promising results. The data from the current investigation suggest that as the course of PD advances, the level of total α-Synuclein in CSF is increased, which may be related to the OD of PD patients.

α-Synuclein might cause damage to synapse function (Mundinano et al., 2011), disrupt signal transduction, and eventually lead to the degeneration and death of neurons in olfaction-related brain regions, such as the olfactory bulb, etc. Further correlation analyses demonstrated that, with α-Synuclein level in CSF elevated, olfactory DIS and ID of PD patients were dramatically damaged, whereas olfactory THR was not significantly impaired (Table 5). Neuropathological results from postmortem studies showed abnormal α-Synuclein deposition in olfaction-related regions of PD brains, such as the olfactory bulb, anterior olfactory nucleus, olfactory tubercles, piriform cortex, anterior area of entorhinal area, and orbitofrontal cortex, etc., especially in the temporal part of the piriform cortex (Silveira-Moriyama et al., 2009; Duda, 2010). However, in the peripheral part of the olfactory pathway, such as the olfactory cells and their nerves, abnormal α-Synuclein deposition or LBs formation was not found. α-Synuclein expressed in olfactory mucosa among individuals with PD and other neurodegenerative diseases as well as normal elderly people were not significantly different (Duda et al., 1999). Thus, α-Synuclein might mainly cause damage to the function of the olfactory cortex, such as DIS and ID, but not to the function of peripheral olfactory structure, such as THR.

**Table 5:** Correlation analyses between the levels of neuropathologic proteins and the score of olfactory function in PD patients.

	**α-Synuclein**		**Aβ1-42**		**T-tau**	
	**r**	***P***	**r**	***P***	**r**	***P***
TDI	−0.261	0.030^*^	−0.205	0.107	0.132	0.329
THR	−0.138	0.26	−0.047	0.716	0.157	0.243
DIS	−0.262	0.030^*^	−0.105	0.412	0.267	0.041^*^
ID	0.307	0.010^*^	−0.259	0.037^*^	−0.53	0.695

* *P <0.05; TDI, threshold, discrimination and identification; THR, threshold; DIS, discrimination; ID, identification; Aβ, β amyloid; T-tau, total tau; PD-OD, Parkinson disease with olfactory dysfunction; PD-NOD, Parkinson disease without olfactory dysfunction*.

Aβ pathology was reported to be associated with non-motor symptoms of PD. Aβ level in CSF was related to memory impairment in PD patients with dementia (PDD) (Mollenhauer et al., 2006). In a 3-year prospective study for PD patients, when the number of correct answers of six odors (lumber, menthol, Japanese orange, gas for household use, Hinoki cypress, and condensed milk) by Open Essence and Jet Stream Olfactometry was four or less, there was a possibility that MMSE declined in 3 years (Fujio et al., 2019). Aβ 1-42 levels in CSF from PD patients with fatigue were significantly lower than those without fatigue (Zuo et al., 2016). Cognitive decline occurs with prolonged disease duration. Increasingly activated microglia produce a large amount of neuroinflammatory factors and subsequently initiate the formation and deposition of Aβ1-42 in PD brains, causing the decrease of Aβ1-42 in CSF. Different from cognitive decline, olfactory dysfunction is the earliest symptom according to the Braak stage of PD pathology. Moreover, PD patients recruited in this study were in an earlier stage of disease with the average duration of 3 years. It was found that Aβ1-42 level in CSF from the PD-OD group was elevated, indicating a smaller deposition of Aβ1-42 in the brain of the PD-OD group. We speculated that the increase of Aβ1-42 may be compensation for the patients in the early stage of PD. Although results from this investigation (Table 4) did not support the finding reported by a previous study showing a decline of Aβ1-42 in CSF from PD patients with worse olfactory function by UPSIT, Aβ1-42 level in CSF was significantly and negatively correlated with ID score, suggesting that ID worsened as Aβ1-42 level in CSF increased (Table 5). The data imply a potential role for Aβ1-42 in the olfactory function of PD. Different from other non-motor symptoms of PD, Aβ1-42 level in CSF was increased in OD, this mechanism needs further investigation.

tau contributes to the integrity of the cytoskeleton under normal conditions. Elevation of T-tau is indicative of axonal damage in AD patients. P-tau mainly includes P-tau (S199), P-tau (S396), P-tau (T181), and P-tau (T231). Excessive P-tau results in the impairment of cell integrity, loss of physiological function, and the eventual degeneration and death of neurons, thus playing an important role in neurodegenerative diseases, such as PD. It was reported that tau was increased in PDD patients (Andreasson et al., 2007). However, in PD patients at an early stage, the T-tau level and the P-tau181 level in CSF was significantly declined (Kang et al., 2013). It was observed that Ta1-3RT transgenic mice over-expressing tau in the olfactory bulb exhibited evident OD (Gill et al., 2008). Additionally, an investigation reported that tau level in the anterior olfactory nucleus was associated with LB formation in the amygdalae nucleus and entorhinal cortex, both of which are pivotal olfactory regions in brain (Takeda et al., 2014). The above data indicate that tau might be related to PD-OD. There was no investigation into the correlation between OD and the levels of T-tau and multiple forms of P-tau in CSF. Here, compared with the PD-NOD group, the T-tau level in CSF from the PD-OD group significantly declined (Table 4), and the decreased T-tau level in CSF was significantly and positively correlated with the declination of DIS (Table 5), implying that the decrease of T-tau in CSF might result from the increase of T-tau in the brain and lead to DIS. We failed to discover that OD was related to the levels of P-tau (S199), P-tau (S396), P-tau (T181), and P-tau (T231) in CSF from PD patients (Table 4). This might suggest that P-tau-containing neurofibrillary tangles were not the neuropathological mechanism of PD-OD, or the sample size was not sufficient.

A limitation of this study is the relatively insufficient CSF samples. It was difficult to obtain CSF from PD patients who were old, combined with low intracranial pressure, spinal deformity, and bone hyperplasia, etc. We will be collecting CSF from PD patients to confirm the conclusion of this study in the future.

In summary, PD patients have a high frequency of OD, objective testing by Sniffin' Sticks is valuable for identifying more PD patients with OD, as most PD patients have significantly impaired olfactory function before the onset of motor symptoms. The OD of PD patients includes impairments of olfactory THR, DIS, and ID. PD-OD may be correlated with the elevation of α-Synuclein and the decrease of T-tau in CSF. Results from this investigation indicate the clinical features and potential neuropathologic mechanism of PD-OD.

## Data Availability Statement

The original contributions presented in the study are included in the article/supplementary materials, further inquiries can be directed to the corresponding author/s.

## Ethics Statement

The studies involving human participants were reviewed and approved by ethics review board of Beijing Tiantan Hospital, Capital Medical University. The patients/participants provided their written informed consent to participate in this study.

## Author Contributions

WZ: study concept and design, acquisition, analysis and interpretation of data, editing and critical revision of the manuscript, and study supervision. PG: acquisition and analysis of data, and study supervision. R-dW: acquisition and analysis of data, and manuscript editing. T-hL: acquisition and analysis of data. D-yD, W-JZ, J-hL, and H-yG: analysis of data and manuscript editing. D-nL, S-yY, LL, YH, L-jZ, and Q-jY: data acquisition. D-nL: data acquisition. L-xL: patient recruitment. X-mW: study design and supervision. All authors contributed to the article and approved the submitted version.

## Conflict of Interest

The authors declare that the research was conducted in the absence of any commercial or financial relationships that could be construed as a potential conflict of interest.

## References

[B1] Andreasson U. Portelius E. Andersson M. E. Blennow K. Zetterberg H. (2007). Aspects of beta-amyloid as a biomarker for alzheimer's disease. Bio Med. 1, 59–78. 10.2217/17520363.1.1.59 20477461

[B2] Braak H. Del Tredici K. Rub U. de Vos R. A. Jansen Steur E. N. Braak E. (2003). Staging of brain pathology related to sporadic Parkinson's disease. Neurobiol Aging. 24, 197–211. 10.1016/S0197-4580(02)00065-9 12498954

[B3] Caggiu E. Arru G. Hosseini S. Niegowska M. Sechi G. Zarbo I. R.. (2019). Inflammation, infectious triggers, and Parkinson's disease. Front Neurol. 10, 122. 10.3389/fneur.2019.00122 30837941 PMC6389614

[B4] Doty R. L. (2012). Olfaction in Parkinson's disease and related disorders. Neurobiol Dis. 46, 527–552. 10.1016/j.nbd.2011.10.026 22192366 PMC3429117

[B5] Duda J. E. (2010). Olfactory system pathology as a model of lewy neurodegenerative disease. J Neurol Sci. 289, 49–54. 10.1016/j.jns.2009.08.042 19783257

[B6] Duda J. E. Shah U. Arnold S. E. Lee V. M. Trojanowski J. Q. (1999). The expression of alpha-, beta-, and gamma-synucleins in olfactory mucosa from patients with and without neurodegenerative diseases. Exp Neurol. 160, 515–522. 10.1006/exnr.1999.7228 10619569

[B7] Ferrer I. Lopez-Gonzalez I. Carmona M. Dalfo E. Pujol A. Martinez A. (2012). Neurochemistry and the non-motor aspects of pd. Neurobiol Dis. 46, 508–526. 10.1016/j.nbd.2011.10.019 22737710

[B8] Fujio H. Inokuchi G. Kuroki S. Tatehara S. Katsunuma S. Kowa H.. (2019). Three-year prospective study on olfaction of patients with Parkinson's disease. Auris, Nasus, Larynx. 47, 899–904. 10.1016/j.anl.2019.08.008 31506174

[B9] Fullard M. E. Morley J. F. Duda J. E. (2017). Olfactory dysfunction as an early biomarker in Parkinson's disease. Neurosci Bull. 33, 515–525. 10.1007/s12264-017-0170-x 28831680 PMC5636737

[B10] Fullard M. E. Tran B. Xie S. X. Toledo J. B. Scordia C. Linder C.. (2016). Olfactory impairment predicts cognitive decline in early Parkinson's disease. Parkinson Rel Disord. 25, 45–51. 10.1016/j.parkreldis.2016.02.013 26923521 PMC4825674

[B11] Gill D. J. Freshman A. Blender J. A. Ravina B. (2008). The montreal cognitive assessment as a screening tool for cognitive impairment in Parkinson's disease. Mov Disord. 23, 1043–1046. 10.1002/mds.22017 18381646

[B12] Goris A. Williams-Gray C. H. Clark G. R. Foltynie T. Lewis S. J. Brown J.. (2007). Tau and alpha-synuclein in susceptibility to, and dementia in, Parkinson's disease. Ann Neurol. 62, 145–153. 10.1002/ana.21192 17683088

[B13] Haehner A. Boesveldt S. Berendse H. W. Mackay-Sim A. Fleischmann J. Silburn P. A.. (2009). Prevalence of smell loss in Parkinson's disease–a multicenter study. Parkinson Rel Disord. 15, 490–494. 10.1016/j.parkreldis.2008.12.005 19138875

[B14] Haehner A. Hummel T. Hummel C. Sommer U. Junghanns S. Reichmann H. (2007). Olfactory loss may be a first sign of idiopathic Parkinson's disease. Mov Dis. 22, 839–842. 10.1002/mds.21413 17357143

[B15] Hummel T. Kobal G. Gudziol H. Mackay-Sim A. (2007). Normative data for the “sniffin' sticks” including tests of odor identification, odor discrimination, and olfactory thresholds: an upgrade based on a group of more than 3,000 subjects. Eur Arch Oto-Rhino-Laryngol. 264, 237–243. 10.1007/s00405-006-0173-0 17021776

[B16] Kang J. H. Irwin D. J. Chen-Plotkin A. S. Siderowf A. Caspell C. Coffey C. S.. (2013). Association of cerebrospinal fluid beta-amyloid 1-42, t-tau, p-tau181, and alpha-synuclein levels with clinical features of drug-naive patients with early Parkinson disease. JAMA Neurol. 70, 1277–1287. 10.1001/jamaneurol.2013.3861 23979011 PMC4034348

[B17] Khoo T. K. Yarnall A. J. Duncan G. W. Coleman S. O'Brien J. T. Brooks D. J.. (2013). The spectrum of nonmotor symptoms in early Parkinson's disease. Neurology. 80, 276–281. 10.1212/WNL.0b013e31827deb74 23319473 PMC3589180

[B18] Kim C. Ho D. H. Suk J. E. You S. Michael S. Kang J.. (2013). Neuron-released oligomeric α- synuclein is an endogenous agonist of TLR2 for paracrine activation of microglia. Nat Commun. 4, 1562. 10.1038/ncomms2534 23463005 PMC4089961

[B19] Lee H. J. Suk J. E. Bae E. J. Lee S.-J. (2008). Clearance and deposition of extracellular alpha-synuclein aggregates in microglia. BiochemBiophys Res Commun. 372, 423–8. 10.1016/j.bbrc.2008.05.045 18492487

[B20] Leonhardt B. Tahmasebi R. Jagsch R. Pirker W. Lehrner J. (2019). Awareness of olfactory dysfunction in Parkinson's disease. Neuropsychology. 33, 633–641. 10.1037/neu0000544 30945913

[B21] Lindstr m V. Gustafsson G. Sanders L. H. Howlett E. H. Sigvardson J. Kasrayan A.. (2017). Extensive uptake of α-synuclein oligomers in astrocytes results in sustained intracellulardeposits and mitochondrial damage. Mol Cell Neurosci. 82, 143–56. 10.1016/j.mcn.2017.04.009 28450268

[B22] Mahlknecht P. Pechlaner R. Boesveldt S. Volc D. Pinter B. Reiter E.. (2016). Optimizing odor identification testing as quick and accurate diagnostic tool for Parkinson's disease. Mov Disord. 31, 1408–1431. 10.1002/mds.26637 27159493 PMC5026160

[B23] Mollenhauer B. Trenkwalder C. von Ahsen N. Bibl M. Steinacker P. Brechlin P.. (2006). Beta-amlyoid 1-42 and tau-protein in cerebrospinal fluid of patients with Parkinson's disease dementia. Dem Geriat Cogn Disord. 22, 200–208. 10.1159/000094871 16899997

[B24] Mundinano I. C. Caballero M. C. Ordonez C. Hernandez M. DiCaudo C. Marcilla I.. (2011). Increased dopaminergic cells and protein aggregates in the olfactory bulb of patients with neurodegenerative disorders. Acta Neuropathol. 122, 61–74. 10.1007/s00401-011-0830-2 21553300

[B25] Parnetti L. Chiasserini D. Persichetti E. Eusebi P. Varghese S. Qureshi M. M.. (2014). Cerebrospinal fluid lysosomal enzymes and alpha-synuclein in Parkinson's disease. Mov Disord. 29, 1019–1027. 10.1002/mds.25772 24436092 PMC4282452

[B26] Parnetti L. Gaetani L. Eusebi P. Paciotti S. Hansson O. El-Agnaf O.. (2019). CSF and blood biomarkers for Parkinson's disease. Lancet Neurol. 18, 573–586. 10.1016/S1474-4422(19)30024-9 30981640

[B27] Ponsen M. M. Stoffers D. Wolters E. Booij J. Berendse H. W. (2010). Olfactory testing combined with dopamine transporter imaging as a method to detect prodromal Parkinson's disease. J Neurol Neurosurg Psychiatry. 81, 396–399. 10.1136/jnnp.2009.183715 19965851

[B28] Pont-Sunyer C. Hotter A. Gaig C. Seppi K. Compta Y. Katzenschlager R.. (2015). The onset of nonmotor symptoms in Parkinson's disease (the onset pd study). Mov Disord. 30, 229–237. 10.1002/mds.26077 25449044

[B29] Postuma R. B. Berg D. Stern M. Poewe W. Olanow C. W. Oertel W. (2015). Mds clinical diagnostic criteria for Parkinson's disease. Mov Dis. 30, 1591–1601. 10.1002/mds.26424 26474316

[B30] Silveira-Moriyama L. Holton J. L. Kingsbury A. Ayling H. Petrie A. Sterlacci W.. (2009). Regional differences in the severity of lewy body pathology across the olfactory cortex. Neurosci Lett. 453, 77–80. 10.1016/j.neulet.2009.02.006 19356597

[B31] Stefanova N. Fellner L. Reindl M. Masliah E. Poewe W. Wenning G. K.. (2011). Toll -like receptor 4 promotes α-synuclein clearance and survival of nigral dopaminergic neurons. Am J Pathol. 179, 954–63. 10.1016/j.ajpath.2011.04.013 21801874 PMC3157205

[B32] Takeda A. Baba T. Kikuchi A. Hasegawa T. Sugeno N. Konno M.. (2014). Olfactory dysfunction and dementia in Parkinson's disease. J Parkinson's Dis. 4, 181–187. 10.3233/JPD-130277 24625830

[B33] Uytingco C. R. Green W. W. Martens J. R. (2019). Olfactory loss and dysfunction in ciliopathies: molecular mechanisms and potential therapies. Curr Med Chem. 26, 3103–3119. 10.2174/0929867325666180105102447 29303074 PMC6034980

[B34] Whitcroft K. L. Cuevas M. Haehner A. Hummel T. (2017). Patterns of olfactory impairment reflect underlying disease etiology. Laryngoscope. 127, 291–295. 10.1002/lary.26229 27556251

[B35] Wilson R. S. Yu L. Schneider J. A. Arnold S. E. Buchman A. S. Bennett D. A. (2011). Lewy bodies and olfactory dysfunction in old age. Chem Sen. 36, 367–373. 10.1093/chemse/bjq139 21257733 PMC3073534

[B36] Witt M. Bormann K. Gudziol V. Pehlke K. Barth K. Minovi A.. (2009). Biopsies of olfactory epithelium in patients with Parkinson's disease. Mov Dis. 24, 906–914. 10.1002/mds.22464 19205070

[B37] Zuo L. J. Yu S. Y. Wang F. Hu Y. Piao Y. S. Du Y.. (2016). Parkinson's disease with fatigue: clinical characteristics and potential mechanisms relevant to alpha-synuclein oligomer. J Clin Neurol. 12, 172–180. 10.3988/jcn.2016.12.2.172 26869370 PMC4828563

